# Circulating microRNA-126 in patients with coronary artery disease: correlation with LDL cholesterol

**DOI:** 10.1186/1477-9560-10-16

**Published:** 2012-08-28

**Authors:** Xiao Sun, Man Zhang, Akimasa Sanagawa, Chieko Mori, Shiori Ito, Soichiro Iwaki, Hiroki Satoh, Satoshi Fujii

**Affiliations:** 1 Division of Cardiovascular Medicine, FengTian Hospital, Shenyang 110024, China; 2 Department of Molecular and Cellular Pathobiology and Therapeutics, Graduate School of Pharmaceutical Sciences Nagoya City University, Nagoya 4678603, Japan; 3 Department of Clinical Management and Informatics, Hokkaido Information University, Ebetsu 0698585, Japan

**Keywords:** Coronary artery disease, Circulating microRNA, LDL cholesterol

## Abstract

**Background:**

Coronary artery disease (CAD) is a major problem worldwide. Atherosclerosis and thrombosis underlying CAD involve multiple cell types. New and useful diagnostic markers are required. MicroRNAs (miRNAs) are a class of noncoding RNAs that posttranscriptionally regulate the gene expressions involved in various cellular processes. Endothelial dysfunction is implicated in early processes of athero-thrombosis. Thus, it was hypothesized that the level of vascular endothelium-enriched miRNAs would be altered in plasma samples of CAD patients.

**Methods:**

Vascular endothelium-enriched miRNA (miR-126) level was analyzed in plasma from 31 patients with CAD and 36 patients without CAD (qRT-PCR analysis).

**Results:**

MiR-126 was not significantly down-regulated or up-regulated in CAD patients. Interestingly, the level of miR-126 was significantly decreased in patients with CAD and high low-density lipoprotein (LDL) cholesterol level. In contrast, the level of miR-126 was significantly increased when LDL cholesterol was high in patients who had risk factors for CAD but did not have angiographically significant CAD.

**Conclusion:**

MiR-126 was not significantly down-regulated or up-regulated in CAD patients and was not suitable for discriminating CAD patients from patients without CAD. The oppositely-directed relationship between miR-126 and LDL cholesterol in patients with or without CAD may have significant implications for identifying a potential role of miR-126 in cholesterol metabolism.

## Background

Coronary artery disease (CAD) is a major problem worldwide including Asian countries. Atherosclerosis and thrombosis underlying CAD involves multiple cell types [1]. Various blood markers associated with CAD have been identified, but only a few have a diagnostic impact or important clinical implications that affect management [2]. Therefore, biomarkers that can assess the risk for CAD and the early atherosclerotic process would be desirable. Circulating microRNAs (miRNAs) could be useful as biomarkers for various diseases including cancer [3], acute myocardial infarction [4], vascular diseases [5] and heart failure [6]. MiRNAs are a class of short (20-25 nt) single-stranded noncoding RNAs that regulate cellular functions through degradation and translational repression of mRNAs that contain complementary sequences. More than 1,000 human miRNAs are identified. In tissue miRNAs regulate the expression of genes involved in differentiation, growth, proliferation and apoptosis. MiRNAs regulate protein expressions as well [7]. MiRNAs are found in tissues, plasma and other body fluids in a stable form that is protected from endogenous RNase activity [3]. Thus, the signatures of miRNA in tissues and plasma may have a potential role in diagnosis, therapeutic efficacy and prognosis. In this study miRNA level in plasma of patients with angiographically significant CAD was compared to that of patients without angiographically significant CAD. Because endothelial dysfunction is involved in early processes of atherosclerosis and thrombosis [8], vascular endothelium-enriched miRNA (miR-126) [9] that can regulate angiogenesis was analyzed.

## Materials and methods

### Study population

Study participants were enrolled from FengTian Hospital, consisting of 67 consecutive patients enrolled prior to undergoing elective cardiac catheterization between January, 2011 and October, 2011. Patients aged 40–81 years were interviewed to collect medical history and lifestyle habits. Risk factors were determined by physician diagnosis and/or treatment for hypertension, hyperlipidemia and diabetes. Coronary angiograms were evaluated independently by 28 operators who made visual estimation of luminal narrowing in multiple segments based on the AHA/ACC classification of the coronary tree. Significant CAD was defined as at least one major epicardial vessel with >50% stenosis. Patients with spastic angina were not included. The study was approved by the Institutional Review Board. All patients provided informed consent at the time of enrollment.

### RNA isolation

Venous blood samples (5 ml) were collected via antecubial venipuncture in EDTA containing tubes. Blood was rapidly centrifuged (1,200 x g for 10 min at 4°C). Supernatant was collected and centrifuged (12,000 x g for 10 min at 4°C). Plasma was obtained and 250 μl was rapidly subjected to RNA extraction. MiRNA was isolated using TRIzol LS RNA isolation kit (Invitrogen, Tokyo, Japan) according to the manufacturer’s protocol and immediately stored at -80°C.

### miRNA qRT-PCR

With 10 ng total RNA miRNA reverse transcription was performed using the TaqMan microRNA Reverse transcription Kit (Applied Biosystems) at 16°C for 30 min, 42°C for 30 min and denaturation of the enzyme at 85°C for 5 min. A Real Time PCR Assay Kit (Applied Biosystems) was used to detect miRNA levels. Each reaction was carried out in a total volume of 20 μl containing: 2 μl RT product, 1.0 μl 20x TaqMan Micro RNA assay primer, 10 μl 2× TaqMan Universal PCR Master Mix and nuclease-free H_2_O to adjust the volume. The PCR reaction was performed as follows; stage 1, 95°C for 10 min, stage 2, 95°C for 15 s, 60°C for 1 min. Stage 2 was repeated for 60 cycles. Real time PCR was performed with the Applied Biosystems 7300 Real-Time PCR Systems at the 9600 emulation run mode. Ct values were normalized to those of miR-16 [10-12].

### Statistical analysis

Statistical analyses were performed using SPSS software (IBM, Tokyo, Japan). Values are expressed as means ± standard deviation (SD). Continuous variables were compared using the Mann–Whitney *U* test or the Kruskall-Wallis test. For categorized values comparisons were made by ANOVA and for post-hoc confirmation, Bonferoni test was used. Linear regression analysis was used to compare plasma miR-126 and LDL cholesterol, and Kendall τb correlation was calculated to evaluate the correlation between the data. A p value <0.05 (two tailed) was considered significant.

## Results

**Figure 1:**
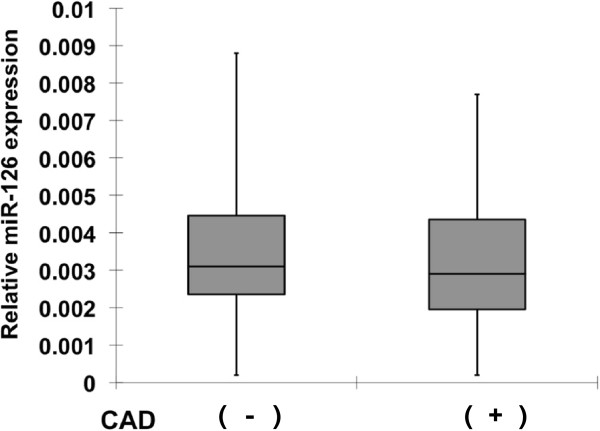
**Box plots of plasma level of miR-126 in patients with angiographically significant CAD (n = 31) and patients without CAD (n = 36).** TaqMan miRNA assays were performed on RNA isolated from plasma obtained from the 2 different groups. No significant difference was observed about the plasma miR-126 levels (p = 0 .675). The level of plasma miR-126 is normalized to miR-16. The lines inside the boxes denote the medians. Mann-Whitey *U* test or Kuskall-Wallis test was used to determine statistical significance.

The clinical characteristics of patients registered are shown in Table 1. Among 67 patients who had risk factors for CAD, 31 patients were found to have angiographically significant CAD and 36 patients were found not to have angiographically significant CAD. Patients who have angiographically significant CAD exhibited higher brain natriuretic peptide (BNP) and New York Heart Association (NYHA) classification. The prevalence of hypertension (50% in patients without CAD vs 60% in patients with CAD) and diabetes (30% vs 25%) were not different between the 2 groups. Patients who do not have angiographically significant CAD exhibited lower prevalence of dyslipidemia (35% vs. 55%). The lipid profiles were similar between the 2 groups due to medications. Patients who do not have angiographically significant CAD exhibited less frequent use of aspirin (25% vs. 95%), β blockers (0% vs. 85%), angiotensin-converting enzyme inhibition/angiotensin receptor blockers (20% vs. 90%), statin (0% vs. 90%), long acting nitrates (0% vs. 85%), insulin (0% vs. 15%), and sulfonyl urea (5% vs. 15%). Use of calcium channel blocker was similar (30% vs. 20%). The miRNA levels in blood are low and microarray approach may lack the sensitivity to identify miRNAs that might serve as disease biomarkers. Thus, qRT-PCR was performed on RNA isolated from plasma of 31 patients with angiographically significant CAD and 36 patients who had risk factors for CAD but did not have angiographically significant CAD. The level of miRNA that was reported to be relatively abundantly present in plasma and tended to have different levels in type 2 diabetes (9) was evaluated. This analysis included miR-126 that was not significantly down-regulated in the blood of patients with CAD as compared to patients without CAD (Figure 1), suggesting that this miRNA is not a marker specific for significant CAD.

**Table 1:** Characteristics of the patients subjected to coronary angiography

**Variables**	**CAD (-)**	**CAD (+)**	**P value**
**n**	**36**	**31**	
Age (year)	61 ± 7	64 ± 11	0.11
BMI (kg/m^2^)	25.6 ± 2.8	24.6 ± 2.3	0.12
Systolic BP (mmHg)	134 ± 16	138 ± 21	0.44
Diastolic BP (mmHg)	83 ± 12	85 ± 15	0.50
Plasma Glucose (mg/dl)	114 ± 48	112 ± 23	0.86
HbA1c (%)	6.2 ± 1.4	5.7 ± 1.3	0.11
Total cholesterol (mg/dl)	188 ± 40	170 ± 44	0.09
LDL cholesterol (mg/dl)	119 ± 30	113 ± 38	0.48
HDL cholesterol (mg/dl)	45 ± 18	43 ± 15	0.61
Triglyceride (mg/dl)	138 ± 73	141 ± 89	0.88
BNP (pg/ml)	16 ± 37	105 ± 144	<0.001
NYHA class	1.0 ± 0	1.7 ± 1.0	<0.001

Data are expressed as means ± SD. A p value <0.05 was considered significant. *BMI* body mass index, *BP* blood pressure. *LDL* low-density lipoprotein, *HDL* high-density lipoprotein, *BNP* brain natriuretic peptide, *NYHA* New York Heart Association.

**Figure 2:**
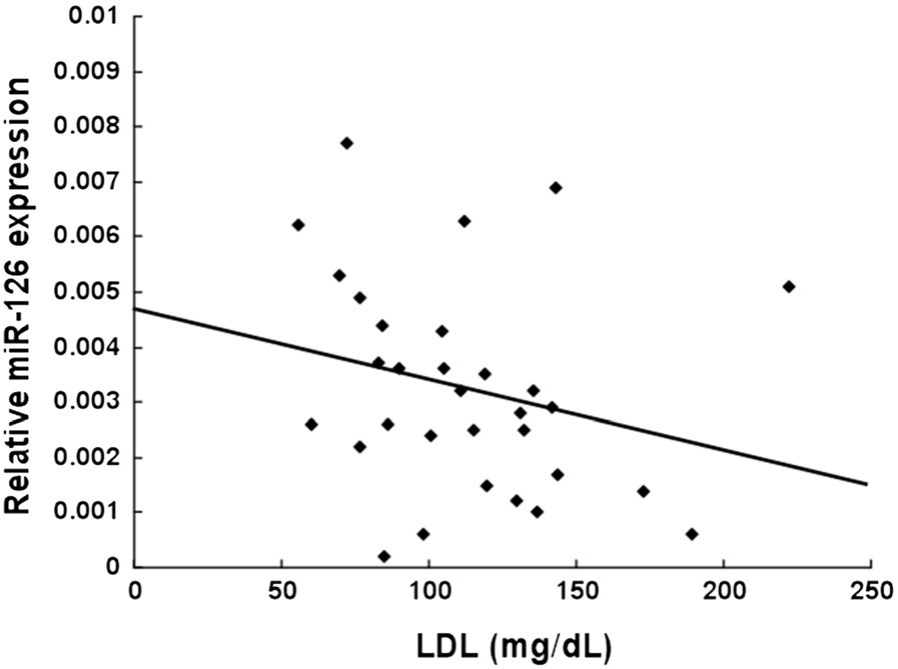
**The correlation between LDL cholesterol and the circulating levels of miR-126 in patients with angiographically significant CAD was analyzed.** Circulating levels of miR-126 were decreased in CAD patients who were showing higher levels of LDL cholesterol (n = 31). Significant negative relationship was observed (R^2^ = 0.059). Kendall τb (coefficient of correlation) = -0.253, significance probability p = 0.047.

**Figure 3:**
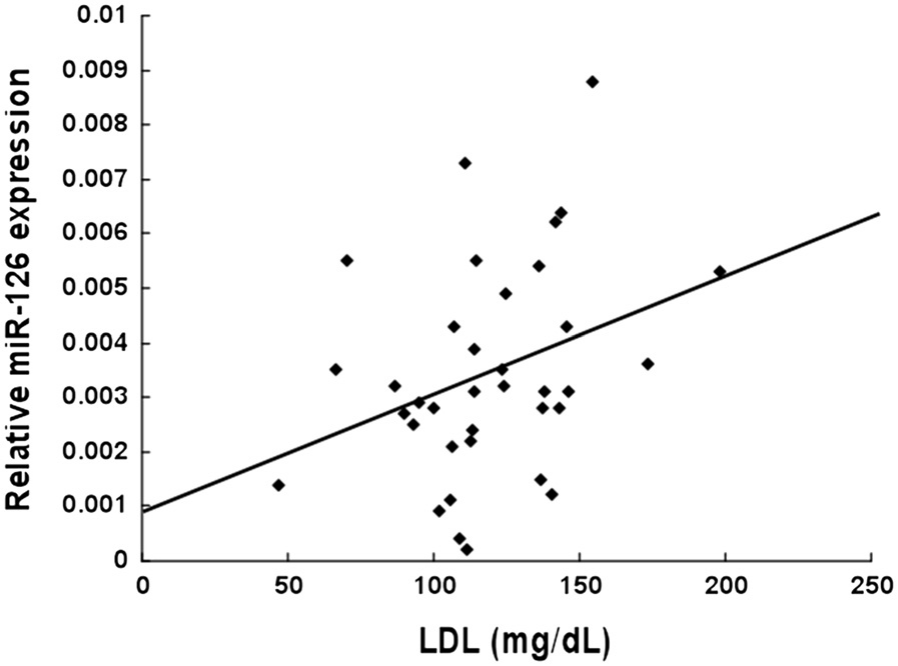
**The correlation between LDL cholesterol and the circulating levels of miR-126 in patients with CAD risk factors but no significant CAD lesions was analyzed.** Circulating levels of miR-126 were increased in patients without CAD who were showing higher levels of LDL cholesterol (n = 36). Significant positive relationship was observed (R^2^ = 0.1055). Kendall τb (coefficient of correlation) =0.245, significance probability p = 0.037.

There has been no report that miR-126 level can be influenced by LDL cholesterol. Interestingly, the level of miR-126 was significantly decreased in patients with CAD and high LDL cholesterol level (Figure 2). In contrast, the level of miR-126 was significantly increased when LDL cholesterol was high in patients who had risk factors for CAD but did not have angiographically significant CAD (Figure 3). These results may imply a link between dysregulation of plasma miR-126 and elevated levels of LDL cholesterol in CAD patients.

## Discussion

Recent studies indicated that circulating miRNA may become valuable biomarkers for different diseases. In this study, it was hypothesized that the plasma miRNA levels could predict the presence of significant CAD. A previous report showed differences in circulating miRNA levels of patients with CAD compared to those of healthy subjects [5]. In this study miR-126 was not down-regulated in CAD patients. Care was taken to stabilize RNA immediately after sample collection and to facilitate storage of the samples for a relatively long period of time without compromising RNA integrity. The plasma concentration of miR-126 was negatively correlated with age and NYHA class, and could be a useful biomarker for heart failure [6]. In this study there were no significant differences in age between the 2 groups (Table 1). Although CAD patients had slightly higher proportion of patients with high NYHA class, the miR-126 levels did not differ. Frequent use of medications in CAD patients did not affect miR-126 levels, either.

mRNA profiling of blood or peripheral mononuclear cells has been applied to cardiovascular diseases [13], and a relationship has been identified between mRNA levels in whole blood and extent of CAD. The present study extends this work by providing insight into the plasma level of miR-126 that is potentially involved in regulating CAD. Because the present analysis included relatively small sample size and microarray method may exhibit reduced sensitivity, sensitive qRT-PCR was used. The level of miR-126 was normalized to that of miR-16, which is highly and consistently expressed across blood samples from patients and controls [10-12,14]. Analysis with qRT-PCR of plasma samples revealed similar levels of miR-126 with or without CAD. Two possible reasons may exist. Plasma miR-126 profile likely reflects extracellular miRNAs and may not fully reflect intracellular miRNAs levels. It is also likely that the patients without CAD could have subclinical vascular inflammation and other pathophysiological alterations similar to those of the CAD patients. Further analysis of plasma miR-126 levels in age-matched healthy subjects are planned in the future.

The mechanism of decreased level of circulating miR-126 in patients with CAD in proportion to the increase in LDL cholesterol remains unclear. Although miRNA is known to be associated with high-density lipoprotein (HDL) cholesterol [15], HDL levels were similar between the 2 groups. It has been hypothesized that the levels of circulating miRNAs are decreased in vascular diseases because they have been taken up into atherosclerotic lesions [5]. The levels of circulating miRNAs may be affected by multiple factors including transcription, processing and stability of the miRNAs within circulating cells, as well as the ability of these cells to release miRNAs into the plasma. Circulating miRNAs may be delivered to cells in the heart or blood vessels through microvesicles, exosomes or apoptotic bodies [16]. Because the present study assessed miR-126 levels in plasma, the present findings may reflect the changes in miRNA transcription or processing as well as the release from the circulating cells. It remains to be determined whether down-regulation of miR-126 in CAD patients is directly involved in inflammation or a compensatory response to this process. Based on the observed changes in miR-126 level in association with LDL cholesterol, the circulating miRNA levels may reflect a compensatory response to inflammation under hyperlipidemic background. During the course of the vascular disease processes endothelial miR-126 may be depleted and plasma levels may start to decrease [9]. The vascular miR-126 is consumed during transcoronary passage [17]. Certain miRNAs are reported to regulate cholesterol efflux [18,19]. However, miR-126 is not predicted to target component(s) of LDL. Although statin was frequently used in CAD patients in this study, statin does not influence miR-126 in endothelial progenitor cells [20]. Further studies are necessary to explore the underlying mechanisms potentially linking LDL cholesterol and circulating miR-126 levels in patients with CAD.

## Conclusion

Although the potential utility of plasma levels of miR-126 as biomarkers for CAD was not demonstrated, the present study provided insight into the potential role of miR-126 in cholesterol metabolism. Further elucidation of the role that miR-126 plays in cholesterol metabolism may contribute to the understanding of the disease process of atherosclerosis and thrombosis.

## Competing interests

The author’s declare that they have no competing interests.

## Authors’ contributions

XS and MZ did all the laboratory work and data collection, AS, CM and SI did all the calculation and contributed in the study design, SI participated in the study design and manuscript preparation, HS was involved in the statistical analysis, SF participated in the design of the study and manuscript preparation. All authors read and approved the manuscript.
